# Mitochondrial apoptosis: facilitator of NK cell-mediated immunotherapy

**DOI:** 10.1038/s41392-022-01126-4

**Published:** 2022-08-18

**Authors:** Evelyn Ullrich, Meike Vogler, Ivana von Metzler

**Affiliations:** 1 University Hospital Frankfurt, Department for Children and Adolescents Medicine, Goethe University, Frankfurt am Main, Germany; 2 Experimental Immunology, Goethe University, Frankfurt am Main, Germany; 3 Frankfurt Cancer Institute, Goethe University, Frankfurt am Main, Germany; 4 University Cancer Center Frankfurt (UCT), University Hospital Frankfurt, Goethe University, Frankfurt am Main, Germany; 5 Institute for Experimental Cancer Research in Pediatrics, Goethe University, Frankfurt, Germany; 6 Department of Medicine, Hematology and Oncology, University Hospital Frankfurt, Goethe University, Frankfurt am Main, Germany

**Keywords:** Cancer therapy, Cancer

In a recent study published in *Cell*, Pan et al. reported how NK cell-based immunotherapy can be combined with BCL2 Homology Domain (BH)3 mimetics to increase tumor cell killing, thus highlighting a novel strategy for cancer immunotherapy.^1^ Induction of mitochondrial apoptosis is a multi-step procedure, and knowledge about the regulation of apoptosis may pave the way for new treatment approaches in immune-oncology.

Despite established chemotherapy protocols and the incorporation of targeted therapies, difficult-to-treat malignancies such as acute myeloid leukemia (AML) and solid tumors still have a high recurrence rate, highlighting the need to develop efficacious non-toxic treatment concepts.

In this context, novel immunotherapy approaches are emerging, including the use of antibodies and cellular therapies. Especially for the treatment of hematological diseases, such as acute B-cell leukemia (B-ALL) and diffuse large B-cell lymphoma (DLBCL), cellular therapy with chimeric antigen receptor (CAR)-engineered autologous T cells has achieved considerable responses, which has led to approval of two CD19-targeted CAR-T cell products in the US and Europe. In addition, the use of natural killer cells (NK cells) for the generation of CAR effector cells has been shown to be safe and effective, and offers potential advantages, particularly since allogeneic NK cells can be safely given to an HLA-mismatched recipient as off-the-shelf therapeutics, which can accelerate application in a particular patient^2^ (for review, see Ref. ^3^).

In their recent study, Pan et al develop a novel approach to immunotherapy.^1^ They found that the induction of mitochondrial apoptosis (mtApoptosis) is a key event regulating NK cell-mediated tumor cell killing. This discovery opens up novel therapeutic approaches by sensitizing tumor cells with mtApoptosis inducers. With the FDA approval of venetoclax (Venclexta®), the first mtApoptosis inducer is clinically available for the treatment of leukemia. Thereby, venetoclax selectively inhibits the anti-apoptotic B-cell lymphoma 2 (BCL2) protein, a key regulator of mtApoptosis. Pan et al. report that treatment of both hematological and solid tumor cells with BH3 mimetics like venetoclax increases NK cell-mediated killing at low effector: target (E:T) ratios. Of note, mainly those cells with some intrinsic sensitivity to BH3 mimetics also displayed synergistic killing with NK cells. The synergy appears to be independent of the BH3 mimetic used, as synergy with NK cell-mediated killing was observed in selected cell lines for the BCL-X_L_ inhibitor A1331852 and the MCL1 inhibitor S63845. This observation is supported by another recent study demonstrating synergy between BH3 mimetics and NK cell-mediated killing in spheroids of pediatric solid tumors.^4^

A requirement for safe and beneficial incorporation of BH3 mimetics into cellular immunotherapy is that the given cells are not functionally impaired or killed by the BH3 mimetics. Importantly, Pan et al show that activation by IL-2 renders NK cells resistant to different BH3 mimetics.^1^ Similarly, a previous report showed comparable protection of NK cells by IL-15.^4^ The molecular mechanism underlying this protection is currently not well described, but may involve the upregulation of several BCL2 proteins upon activation.^1^ Taken together, the combination of BH3 mimetics and NK cells is capable of inducing apoptosis at lower concentrations than either approach alone, and thus may be beneficial in overcoming the toxicity of BH3 mimetics while maintaining efficient tumor cell killing.

**Fig. 1:**
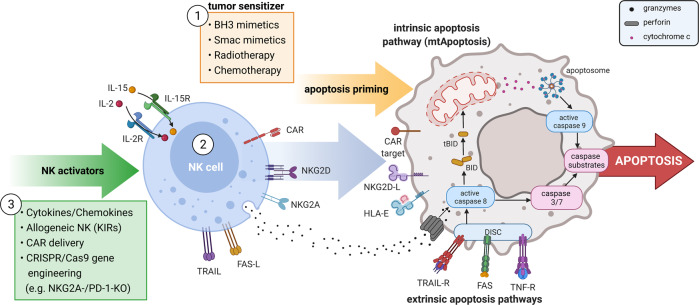
Multi-step scenario of apoptosis-induction in cancer cells. Examples for efficient immune-oncology approaches combining ① tumor sensitizing agents, ② NK cell therapy and ③ NK cell activating strategies. This figure has been created with *BioRender.com*

This study leads to imagine a sophisticated multi-step scenario (Fig. 1) for precise and efficient targeting of cancer cells, based on the knowledge that mtApoptosis plays a central role in NK cell-induced lysis of tumor cells. In perspective, sensitization of tumor cells with BH3 mimetics, efficient pre-activation of NK cells with cytokines, and the implementation of genetically modified NK cells, such as the induction of CARs or CRISPR/Cas9-mediated knockout of immune suppressive checkpoints, could further enhance the cytotoxic potential against malignant cells.

Interestingly, earlier studies described NK cells as “serial killers” based on their capability to lyse more than one tumor cell, but relied on pure observational imaging analysis. Further unraveling of the underlying signaling pathways identified that the first individual target cells are killed by the fast release of cytotoxic granules and afterwards death receptor mediated signaling cascades come into the NK-tumor-interplay.^5^ The findings by Pan et al. now complete the picture with regard to the serial killer capacity of cytokine activated NK cells by demonstrating that final induction of cancer cell death requires more than one hit and that these hits accumulate into the gradual induction of mtApoptosis.

In the perspective for personalized cancer treatment strategies, however, detailed profiling of the patients’ cancer cells will be a key to determine which targeted immune cell therapeutic and which BH3 mimetic should be applied for an individual cancer patient.

As highlighted by the diverse response of tumor cells to the selective BH3 mimetics, the cancer characterization should include biomarkers like BH3 profiling to identify which BH3 mimetics might be the optimal treatment.^1^ In addition, on the side of targeted NK cell therapy optimization, surfaceome analysis should be considered to identify specific target antigens, including possible tumor neo-antigens, but also pathway analysis to unravel the immune inhibitory mechanisms for advanced engineering of the immune cell preparation. Based on these screening analyses and the modular generation of advanced immune cell products, onco-immunotherapy in combination with BH3 mimetics could be extended to a broad panel of difficult-to-treat or therapy-resistant individual cancer entities. Beyond NK cells, it has to be highlighted that relatively little is known to date about the team play of the immune system under treatment with non-toxic doses of BH3 mimetics and adoptive cell therapy, e.g., cytokine-activated and/or engineered NK or T cells, which urgently needs in-depth functional analysis in further studies. We are convinced that the detailed understanding of the resulting immune cell interplay will pave the path to modulate additional help from the patients’ own immune system to finally make this novel treatment approach successful.

These new findings are important for a detailed understanding of apoptosis induction in cancer and lead directly to the next generation of combinatorial onco-immunotherapeutics with the goal of improved personalized cancer therapy. We look forward with excitement and great enthusiasm to the next decade of onco-immunotherapeutics, where it will be important to establish and apply these novel treatment concepts for the benefit of cancer patients.
